# Are Cognitive Changes in Hereditary Spastic Paraplegias Restricted to Complicated Forms?

**DOI:** 10.3389/fneur.2019.00508

**Published:** 2019-05-24

**Authors:** Laís Alves Jacinto-Scudeiro, Gustavo Dariva Machado, Annelise Ayres, Daniela Burguêz, Marcia Polese-Bonato, Carelis González-Salazar, Marina Siebert, Marcondes Cavalcante França Jr., Maira Rozenfeld Olchik, Jonas Alex Morales Saute

**Affiliations:** ^1^Graduate Program in Medicine: Medical Sciences, Universidade Federal Rio Grande do Sul, Porto Alegre, Brazil; ^2^Medical Genetics Service, Hospital de Clínicas de Porto Alegre (HCPA), Porto Alegre, Brazil; ^3^Postgraduate Program in Health Sciences, Universidade Federal de Ciências da Saúde de Porto Alegre, Porto Alegre, Brazil; ^4^Graduate Program in Biochemistry, Federal University of Rio Grande do Sul, Porto Alegre, Brazil; ^5^Graduate program in Medical Physiopathology, School of Medical Science, Universidade Estadual de Campinas, Campinas, Brazil; ^6^Graduate program in Gastroenterology and Hepatology, Universidade Federal do Rio Grande do Sul, Porto Alegre, Brazil; ^7^Unit of Laboratorial Research/Experimental Research Center, Hospital de Clínicas de Porto Alegre, Porto Alegre, Brazil; ^8^Department of Neurology, School of Medical Science, Universidade Estadual de Campinas, Campinas, Brazil; ^9^Department of Surgery and Orthopedics, Faculdade de Odontologia, Universidade Federal do Rio Grande do Sul, Porto Alegre, Brazil; ^10^Department of Internal Medicine, Universidade Federal do Rio Grande do Sul, Porto Alegre, Brazil; ^11^Neurology Service, Hospital de Clínicas de Porto Alegre, Porto Alegre, Brazil

**Keywords:** hereditary spastic paraplegia, HSP, SPG, cognitive profile, memory, executive function

## Abstract

**Background:** Little is known about the cognitive profile of Hereditary Spastic Paraplegias (HSP), where most scientific attention has been given to motor features related to corticospinal tract degeneration.

**Objectives:** We aimed to perform a broad characterization of the cognitive functions of patients with pure and complicated HSP as well as to determine the frequency of abnormal cognitive performances in the studied subtypes.

**Methods:** A two-center cross-sectional case-control study was performed. All individuals underwent cognitive assessment through screening tests (Mini Mental State Examination—MEEM and Montreal Cognitive Assessment—MOCA) and tests to assess specific cognitive functions (Verbal fluency with phonological restriction—FAS; Verbal categorical fluency—FAS-cat and Rey's Verbal Auditory Learning Test -RAVLT).

**Results:** Fifty four patients with genetically confirmed HSP diagnosis, 36 with spastic paraplegia type 4 (SPG4), 5 SPG11, 4 SPG5, 4 cerebrotendinous xanthomatosis (CTX), 3 SPG7 and 2 SPG3A, and 10 healthy, unrelated control subjects, with similar age, sex, and education participated in the study. SPG4 patients had worse performances in MOCA, FAS, FAS-cat, and RAVLT when compared to controls. Most SPG4 patients presented cognitive changes not compatible with dementia, performing poorly in memory, attention and executive functions. SPG5 patients scored lower in executive functions and memory, and SPG7 patients performed poorly on memory tasks. All evaluated cognitive functions were markedly altered in CTX and SPG11 patients.

**Conclusions:** Cognitive abnormalities are frequent in HSP, being more severe in complicated forms. However, cognitive impairments of pure HSPs might impact patients' lives, decreasing families' socioeconomic status and contributing to the overall disease burden.

## Introduction

Hereditary Spastic Paraplegias (HSP) is a heterogeneous group of neurodegenerative genetic diseases (> 80 *loci* have been described) with spastic gait as the core feature (1, 2). HSP subtypes are clinically classified into pure forms—in which only a pyramidal syndrome is found (although changes in vibratory sensation and neurogenic bladder are accepted)—and complicated forms in which pyramidal findings are accompanied by dysfunction in other neurological systems (e.g., ataxia, parkinsonism, cognitive impairment, etc.) or by systemic involvement (3).

Although HSP are rare conditions, recent studies suggest that its prevalence (2–10 per 100,000) is similar to or higher than that of more studied conditions such as hereditary ataxias (4). Spastic paraplegia type 4 (SPG4) is the most frequent autosomal dominant subtype, whereas SPG11 and SPG7 are the most frequent autosomal recessive subtypes of HSP worldwide (4, 5).

There are few studies that have investigated the cognitive profile of HSP patients. Most of then focused on complicated forms, reporting intellectual disability and cognitive deficits perceptible by physician's clinical evaluation in up to 100% of SPG11 patients (6, 7). On the other hand, the cognitive characterization of the pure forms of HSP remains unclear, with some reports describing mild cognitive changes without the use of tests to evaluate specific cognitive functions or only being described through the physician's clinical impression (2, 6–11). Thus, the objective of this study was to characterize the cognitive functions of memory, orientation, verbal fluency, language, attention, and executive functions in HSP patients, to determine the frequency of cognitive changes in the different subtypes, and to correlate these findings with disease severity variables.

## Materials and Methods

### Design and Subjects

We performed a two-center, exploratory, cross-sectional, case-control study conducted at two teaching hospitals in the Brazilian cities of Porto Alegre and Campinas. We included consecutive patients followed at the Neurogenetics outpatient clinics of these hospitals, from December 2016 to August 2018, and who presented genetically confirmed diagnosis of HSP or genetic or biochemical diagnosis of CTX, which can be considered as a complicated form of HSP (5). Healthy, unrelated subjects, with similar sex, age, and education characteristics were recruited from the local community of Porto Alegre as the control group for the adult population. Concomitant neurological or systemic conditions that could present cognitive alterations were exclusion criteria. The project was approved by the institutions' ethics committees under review numbers 170012 and 62653816.7.0000.5404, which follows the Declaration of Helsinki. Informed written consent was obtained from all individuals' prior participation.

### Cognitive Evaluation

Cognitive assessments were performed by a single evaluator (LAJS) through standardized tests validated for the Brazilian population, which analyze a range of competencies, namely:

1. Mini Mental State Examination (MMSE): screening test for cognitive function evaluation. The maximum score is 30 points and, in the Brazilian population, a score of 28 points or more is indicative of normal cognitive function for individuals who have been formally educated for >8 years (12).
2. Montreal Cognitive Assessment (MoCA): screening test for cognitive function evaluation. The maximum score is 30 points and a score of 26 point or more is indicative of normal cognitive function (13).
3. Verbal fluency with phonological restriction (FAS): it consists in naming words beginning with the letters F, A, and S, respectively, and assesses executive function, language and semantic memory. Performance can be affected by education and by age (14).
4. Verbal categorical fluency (animals) (FAS-cat): this measure is a variation of the verbal fluency test and it is restricted to a semantic category. The score may be affected by education (15).
5. Rey's Verbal Auditory Learning Test (RVALT): is a tool to assess immediate memory (RVALT) as well as short (A6) and long-term (A7) retention. The test involves five consecutive repetitions and retrievals of stimuli from a list of 15 words (16).

Cognitive performance for the study population under 17 years-old was evaluated with the Wechsler Intelligence Scale for Children (WISC III) (17). This test can be used as an IQ (Intelligence Quotient) test for children and it is most often used as a clinical tool to measure individual's cognitive abilities. We used the Cubes and Vocabulary subtests to evaluate children's performance. Dementia diagnosis was based on Diagnostic and Statistical Manual of Mental Disorders (DSM-V) (18) criteria.

### Evaluation of Depressive Symptoms

In order to verify if depressive symptoms could act as a confounding factor for cognitive performance, the Beck Depression Inventory (BDI) was applied. BDI is a self-reported questionnaire composed of 21 questions that assess the intensity of depressive symptoms. Scores range from 0 to 63 points that are crescent in severity (19).

### Motor Neurological Evaluation

Neurological severity was assessed by the Brazilian Portuguese version of the Spastic Paraplegia Rating Scale (SPRS). SPRS scores ranges from 0 to 52 and are crescent in severity (20). Disease stage was classified as: (0) asymptomatic; (1) no functional handicap, but signs at examination (slight gait stiffness); (2) mild gait stiffness, walking unlimited, and running still possible; (3) moderate gait stiffness, limited walking without aid, and running impossible; (4) moderate to severe gait stiffness, walking possible with aid; and (5) walking impossible, wheelchair bound. We also estimated the cross-sectional disease progression as the cross-sectional quotient of disease severity (SPRS) and disease duration, as previously established (2). Disease duration and age of onset of the first motor symptom were reported by patients and their relatives.

### Statistical Analysis

Statistical tests were selected according to the distribution of data given by Shapiro-Wilk test and histograms. Descriptive analysis of cognitive assessment scores was carried out based on the tests cut-offs for normal performance according to age and education level in the Brazilian population. Comparisons between SPG4 and controls individuals' scores and between SPG4 patients with truncating and non-truncating variants scores were performed by Student's *t*-test or Mann–Whitney *U*-test. The 95% confidence interval (CI) for differences in means between groups was also provided. Correlations of cognitive performance scores with independent variables (age, education level, age at onset, SPRS, disease stage, disease duration, cross-sectional disease progression) were performed with the Pearson or Spearman correlation tests. A linear regression model was built with independent variables that presented *P* < 0.2 in the simple correlation test, where only variables that maintained *P* < 0.05 after adjustment for covariates were kept in the final model. Statistical significance was defined as *P* < *0.05*.

## Results

A total of 54 patients with HSP were enrolled in the study, including 36 (5 children) SPG4 (17 families), 5 SPG11 (4 families), 4 SPG5 (1 family), 4 CTX (4 families), 3 SPG7 (3 families), and 2 SPG3A (1 family) patients and 10 healthy control subjects. See Table 1 for the clinical and demographical characterization of the study population and Supplementary Table 1 (21–26) for detailed individual data of subjects.

**Table 1:** Demographics of the enrolled individuals.

	**Controls (*N* = 10)**	**SPG4**		**SPG11 (N=5)**	**CTX (*N* = 4)**	**SPG5 (*N* = 4)**	**SPG7 (*N* = 3)**	**SPG3A (*N* = 2)**
		**Adult (*N* = 31)**	**Children (*N* = 5)**					
Female sex	6 (60%)	17 (47%)	2 (40%)	4 (80%)	2 (50%)	2 (50%)	1 (33%)	2 (100%)
Age	46.5 (12.3)	45 (18)	9 (2.9)	36.4 (5.7)	47.5 (10.5)	54 (6.5)	53.3(25.6)	38.5 (17.7)
Educational level	9.4 (3.1)	7.16 (3.9)	3.8 (3.11)	10 (2.8)	8 (4.8)	4.5 (1)	16.3 (1.6)	9 (4.2)
Age at onset	–	34.7 (16.8)	1.75 (1.30)	17.4 (2.8)	25.7 (20.2)	34 (4.9)	21.7(12.5)	11.5 (0.7)
Disease duration	–	16.4 (11.3)	8.2 (2.77)	20.4 (4.2)	22 (16.9)	21.2(5.4)	32.3(17.5)	27 (18.4)
SPRS	–	18.7 (9.8)	11.4 (5.9)	37.2 (6.1)	27 (16.1)	33.2 (11.2)	22.6(10.6)	8.5 (3.5)
Disease stage	–	0–0 (0%)	0–0 (0%)	0–0 (0%)	0–0 (0%)	0–0 (0%)	0–0 (0%)	0–0 (0%)
		1–1 (3%)	1–2 (40%)	1–0 (0%)	1–1 (25%)	1–0 (0%)	1–0 (0%)	1–1 (50%)
		2–5 (16%)	2–3 (60%)	2–0 (0%)	2–1 (25%)	2–0 (0%)	2–0 (0%)	2–0 (0%)
		3–8 (26%)	3–0 (0%)	3–0 (0%)	3–0 (0%)	3–1 (25%)	3–1(33%)	3–1 (50%)
		4 − 15(49%)	4–0 (0%)	4–1 (20%)	4–1 (25%)	4–3 (75%)	4–2(66%)	4–0 (0%)
		5–2 (6%)	5–0 (0%)	5–4 (80%)	5–1 (25%)	5–0 (0%)	5–0 (0%)	5–0 (0%)
Cross-sectional disease progression	–	1.13	1.39	1.82	1.22	1.56	0.48	0.31
BDI	–	8.6 (5.9)	NA	NA	9 (1)	4.75 (3.6)	6.3 (5.5)	7 (1.4)

*Data are shown as means in years (standard deviation), except for sex and disease stage that are shown as frequencies. CTX, cerebrotendinous xanthomatosis; SPRS, Spastic Paraplegia Rating Scale; BDI, Beck Depression Inventory; NA, not available—patients unable to perform this test*.

### Patients' Performance in Relation to Cognitive Test Scores

Table 2 details the cognitive performance of HSP patients and controls. Most SPG4 patients presented cognitive changes not compatible with dementia, performing poorly in memory, attention and executive function. SPG5 patients scored lower in executive functions and memory, and SPG7 patients performed poorly on memory tasks, also presenting cognitive changes not compatible with dementia. All evaluated cognitive functions were altered in patients with CTX (2/4 with dementia) and SPG11 (all with dementia) patients. The 2 patients with SPG3A performed within normal limits on cognitive tests. Of note, SPG11 patients were unable to respond to BDI, because of cognitive impairment.

**Table 2:** Group performances in cognitive tests.

	**Controls (*N* = 10)**	**SPG4 (*N* = 31^a^)**	**SPG5 (*N* = 4)**	**SPG7 (*N* = 3)**	**SPG11 (*N* = 5)**	**CTX (*N* = 4)**	**SPG3 (*N* =)**
MMSE	25 (25–27.25)	26 (23–27)	24 (22.5–27)	28 (27.5–28.5)	18 (14–19)	20 (17.5–25)	29.5 (29–30)
	40%	64%	50%	66%	100%	75%	0%
MOCA	24.9 (±2.84)	19.1 (±4.81)	20.75 (±4.42)	22.33 (±0.57)	6.4 (±3.91)	18.5 (±3.05)	27 (±1.41)
	40%	87%	100%	100%	100%	75%	0%
FAS	31.3 (±9.15)	18.67 (±9.47)	20 (±9.76)	21 (±5.56)	6.6 (±3.97)	23 (±8.96)	31 (±8.48)
	0%	45%	50%	66%	100%	25%	0%
FAS-cat	18.1 (±5.64)	11.77 (±4.71)	13.5 (±2.38)	14 (±2)	4.8 (±2.86)	11.5 (±3.31)	21 (±0)
	0%	41%	0%	33%	100%	25%	0%
RAVLT	39.3 (±7.9)	24.22 (±8.49)	30.75 (±5.43)	25.66 (±2.51)	11.4 (±10.89)	23 (±8.08)	40.5 (±0)
	60%	83%	75%	66%	100%	100%	0%
A6	8.2 (±2.86)	3.93 (±2.26)	6.5 (±4.12)	4 (±1.73)	0.2 (±0.44)	2.75 (±2.98)	9 (±1.41)
	40%	83%	75%	100%	100%	75%	0%
A7	8 (6–10.25)	3 (1.5–4)	2.5 (2–**7**.5)	4 (3–4)	0 (0)	2.5 (1–4.5)	9 (0)
	40%	96%	75%	66%	100%	75%	0%

*Data are shown as means (standard deviation), except for MMSE and A7 that are shown in median (interquartile range), and percentages of altered performances according to the normal standards for the given test. CTX, cerebrotendinous xanthomatosis; FAS, verbal fluency with phonological restriction; FAS-cat, verbal categorical fluency (animals); MMSE, Mini Mental State Examination; MOCA, Montreal Cognitive Assessment; RVALT, A6 and A7, Rey's Verbal Auditory Learning Test*.

a *Only adult patients with SPG4 were considered*.

### Comparative Analysis Between SPG4 and Control Subjects

**Figure 1:**
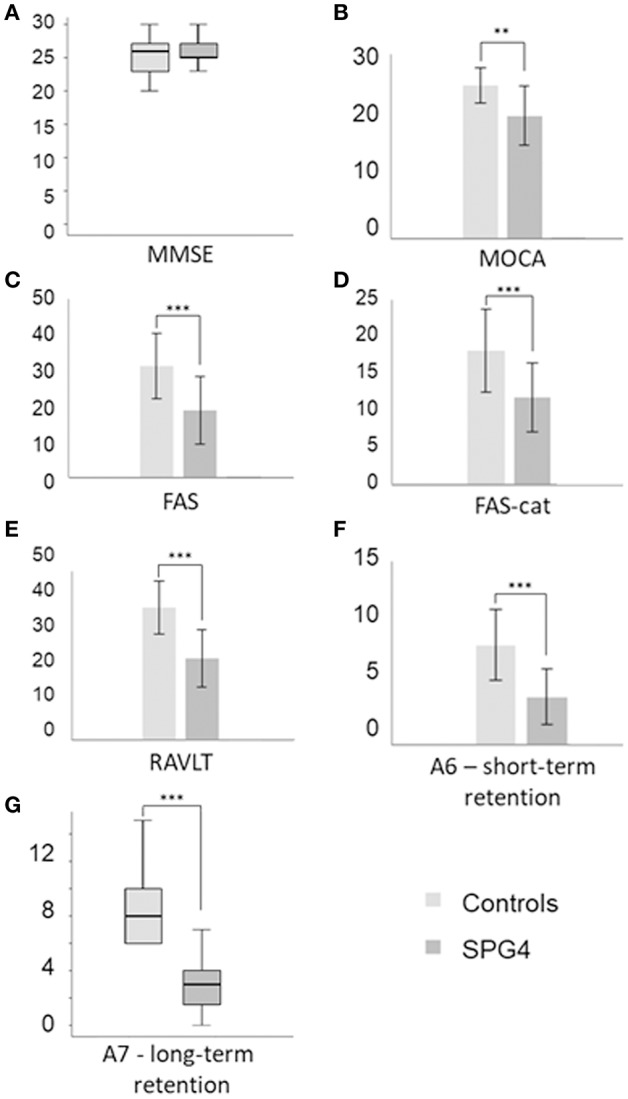
Comparison between control and SPG4 subjects performances in cognitive tests. Bars represent mean values and standard deviation. Box-plots represent median values and interquartile ranges. ^**^P < 0.01 e ^***^P ≤ 0.001. FAS, verbal fluency with phonological restriction; FAS-cat, verbal categorical fluency (animals); MMSE, Mini Mental State Examination; MoCA, Montreal Cognitive Assessment; RVALT, A6 and A7, Rey's Verbal Auditory Learning Test. **(A)** MEEM, **(B)** MOCA, **(C)** FAS, **(D)** FAS-cat, **(E)** RVALT, **(F)** A6 - short-term retention, **(G)** A7 - long-term retention.

While the performance of SPG4 patients was similar to controls in the MMSE (*P* = 0.800, Figure 1A), their scores were lower than those of the control subjects in all other cognitive tests: MOCA (−4.93, 95% CI, −8.20 to −1.66, *P* = 0.004, Figure 1B), FAS (−12.62, 95% CI, −19.54 to −5.70, *P* = 0.001, Figure 1C), FAScat (−6.32, 95% CI, −9.96 to −2.68, *P* = 0.001, Figure 1D), RAVLT (−15.05, 95% CI, −21.22 to −8.92, *P* < 0.001, Figure 1E), A6 (−4.26, 95% CI, −6.04 to −2.48; *P* < 0.001, Figure 1F) and A7 (*P* < 0.001, Figure 1G).

### Which Factors Correlated to Cognitive Decline in SPG4 Patients?

**Figure 2:**
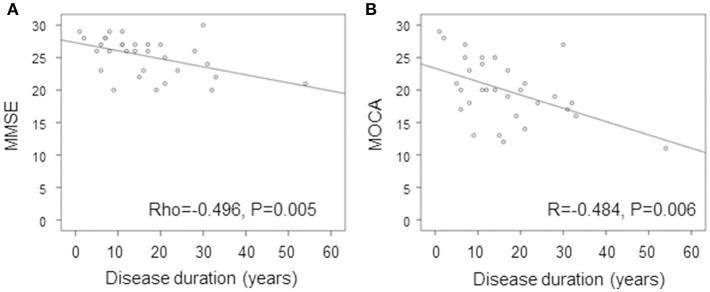
Correlation of **(A)** MMSE and **(B)** MOCA with disease duration in SPG4. Simple correlation of MMSE and MOC with disease duration in SPG4. MMSE, Mini Mental State Examination; MoCA, Montreal Cognitive Assessment.

Although no statistically significant difference was found between SPG4 patients and control subjects in MMSE, both disease duration (Beta = −0.351, 95% CI, −0.170 to −0.014, *R*^2^ = 0.116, *P* = 0.022, Figure 2A) and education years (Beta = 0.492, 95% CI, 0.152 to 0.614, *R*^2^ = 0.227, *P* = 0.002) were independently correlated with MMSE in the linear regression model. For each additional year of disease duration, there was a decrease of 0.092 points in MMSE, and for each additional education year there was an increase of 0.383 points in the MMSE results. Education and disease duration were also the only variables independently correlated in the linear regression model with MOCA. For each additional year of disease duration there was a decrease of 0.162 points in MOCA (Beta = −0.382; 95% CI, −0.294 to −0.030, *R*^2^ = 0.137, *P* = 0.018, Figure 2B), and for each additional education year there was an increase of 0.525 points in MOCA (Beta = 0.416, 95% CI, 0.134 to 0.916, *R*^2^ = 0.163, *P* = 0.01). Age was the only variable correlated with FAS (*R* = −0.520, *P* = 0.003) and RAVLT (*R* = −0.428, *P* = 0.016), and neither significant correlations were found between the independent variables and performances in the FAScat, nor A6 and A7. BDI scores did not correlate with cognitive tests performance (*P* > 0.2 for all comparisons), making it very unlikely that depressive symptoms were influencing the results of the cognitive evaluation.

### Genotype-Phenotype Correlation in SPG4

Variants in *SPAST* were classified as truncating (nonsense and frameshift variants, N = 11 individuals) and non-truncating (missense and in-frame insertion, N = 20 individuals). Clinical, demographical and cognitive findings of patients with truncating and non-truncating variants did not differ; see Supplementary Table 2 for details.

### Cognitive Performance in Children

The WISC-III results showed low average IQ scores in 60% (3/5) of the children with SPG4 and average IQ in the others. There were no children in the other HSP subtypes. Mean cubes subtest (executive subscale) performance was 9.2 (4.16) and mean vocabulary test (verbal subscale) was 7.2 (2.51), which are low average values. All evaluated children had missense pathogenic variants in *SPAST*.

## Discussion

Over the last years, non-motor symptoms of movement disorders have received greater attention (27, 28), albeit in a more timid manner for hereditary spastic paraplegias (29). Classically the pure forms of HSP have been described as solely motor syndromes in which only pyramidal signs are found. However, these descriptions are generally based on the neurologist's impression, without a more formal assessment of non-motor symptoms, such as subtle cognitive changes (2). In this study, we found impairments in memory, attention, executive functions and verbal fluency in SPG4 patients, the most prevalent HSP subtype worldwide and the prototype of pure forms of the disease, and we confirmed the more severe cognitive dysfunction of complicated forms of HSP.

We found cognitive changes not compatible with dementia in most evaluated SPG4 patients, with abnormal cognitive performances ranging from 41 to 96%, depending on the utilized test and domain, with memory (immediate and recent) being the most frequently altered cognitive function. Our results are partially in agreement with descriptions of subtle cognitive deficits in these patients in a few case series (2, 7, 8, 10, 11). The inverse correlation between MMSE and MOCA with disease duration and the normal, albeit low average, performance in children on intelligence tests might suggest that cognitive dysfunction in SPG4 is progressive and worsens with disease progression. SPG4 patients with truncating and with non-truncating variants had similar cognitive performances, which is in agreement with a previous study (8).

Neuroimaging findings from previous studies (30, 31) are in agreement with the cognitive changes we have found, suggesting a more widespread central nervous system involvement in SPG4. Extensive fractional anisotropy reduction in non-motor regions (posterior cingulate gyri and splenium of the corpus callosum) were found in SPG4 patients on magnetic resonance (MRI) diffusion tensor imaging (30), as well as decreased brain activity in the left insular cortex in functional MRI (31), which regulates a wide range of cognitive and emotional functions (32).

Despite the cognitive impairment of SPG4 patients in the overall evaluation, no differences to controls were found with MMSE screening test. On the other hand, most SPG4 patients (87%) scored below normal thresholds for MOCA. The discrepancy of MMSE and MOCA was also verified among patients with SPG5 and SPG7 and was suggested by a previous study, even though this particular tool was not used by that authors (10). Therefore, MOCA is likely to be a more sensitive screening tool for cognitive changes in HSP, especially for pure forms.

Most SPG11 and CTX patients presented major cognitive deficits and scores well below what would be expected for all cognitive functions tested (not only memory). These results corroborate previous findings of severe intellectual disability in this population confirming the higher prevalence and severity of cognitive dysfunction when compared to pure HSP forms (33, 34).

Due to the exploratory nature of the study we neither performed sample size calculation nor definition of the study power and main outcome. Nevertheless, statistically significant differences between SPG4 and controls were found in all tests, except MMSE. There was no trend for lower MMSE scores in SPG4 (*P* = 0.800) when compared to controls and therefore there is a low chance of Type 2 error. Finally, we should mention that we were unable to compare the cognitive performances of children and adults due to the different nature of cognitive tests according to age.

## Conclusion

Cognitive abnormalities are frequent in HSP, with dementia being commonly observed in complicated forms and cognitive changes not compatible with dementia in pure forms of the disease. SPG4, the most frequent and the prototype of pure HSP, present multiple cognitive abnormalities that might impact patients' lives and result in difficulties at school, on their careers and, consequently, might decrease patients' and families' socioeconomic status. Longitudinal studies are needed to assess the rate of progression of cognitive changes and to verify whether motor and cognitive functions have similar or different patterns of progression and thus different pathophysiological processes.

## Ethics Statement

This study was carried out in accordance with the recommendations of Comitê de Ética e Pesquisa-Hospital de Clínicas de Porto Alegre (CEP-HCPA) with written informed consent from all subjects. All subjects gave written informed consent in accordance with the Declaration of Helsinki. The protocol was approved by the Comitê de Ética e Pesquisa (CEP-HCPA).

## Author Contributions

LJ-S, MO, and JS conception and design of the research. LJ-S, AA, GD, DB, MP-B, MS, CG-S, MC, and JS recruitment of patients and data collection. LJ-S and JS tabulation, statistical analysis, creation of tables and figures. LJ-S, MO, and JS writing the text. LJ-S, GD, AA, DB, MP-B, CG-S, MS, MC, MO, and JS review of text and addition of significant parts.

### Conflict of Interest Statement

The authors declare that the research was conducted in the absence of any commercial or financial relationships that could be construed as a potential conflict of interest.
