# Supplementary material for: Are Cognitive Changes in Hereditary Spastic Paraplegias Restricted to Complicated Forms?
Source: Front Neurol. 2019 May 24;10:508. doi: 10.3389/fneur.2019.00508 (PMC6558376; doi:10.3389/fneur.2019.00508)
Supplement: Supplementary file 1 [file Table_1.DOCX]

| **Fam** | **Subject** | **Gene** | **Age** | **Education Level** | **Mutation** | **Mutation Type** | **Variant Classification^a^** | **Age at**  **onset** | **Disease Duration** | **SPRS** | **Disease Stage** |
| --- | --- | --- | --- | --- | --- | --- | --- | --- | --- | --- | --- |
| 1 | HSP1 | *SPAST* | 46 | 10 | c.1492_1493+2delAGGT/- | Deletion | Pathogenic^5^ | 14 | 16 | 29 | 5 |
| 1 | HSP2 | *SPAST* | 49 | 1 | c.1492_1493+2delAGGT/- | Deletion | Pathogenic^5^ | 41 | 9 | 30 | 4 |
| 2 | HSP3 | *SPAST* | 31 | 9 | c.1273G>C/- | Missense | Pathogenic^5^ | 1 | 31 | 8 | 5 |
| 2 | HSP4 | *SPAST* | 11 | 4 | c.1273G>C/- | Missense | Pathogenic^5^ | 1 | 11 | 18 | 2 |
| 2 | HSP5 | *SPAST* | 7 | 2 | c.1273G>C/- | Missense | Pathogenic^5^ | 1 | 8 | 8 | 1 |
| 2 | HSP6 | *SPAST* | 6 | 1 | c.1273G>C/- | Missense | Pathogenic^5^ | 1 | 5 | 12 | 2 |
| 3 | HSP7 | *SPAST* | 36 | 11 | c.1412_1413delinsAC/- | Missense | Pathogenic^5^ | 7 | 30 | 28 | 4 |
| 3 | HSP8 | *SPAST* | 8 | 3 | c.1412_1413delinsAC/- | Missense | Pathogenic^5^ | 4 | 4 | 2 | 0 |
| 4 | HSP9 | *SPAST* | 62 | 14 | c.1378C>T/- | Missense | Pathogenic^5^ | 45 | 14 | 10 | 3 |
| 4 | HSP10 | *SPAST* | 58 | 10 | c.1378C>T/- | Missense | Pathogenic^5^ | 62 | 2 | 6 | 3 |
| 4 | HSP11 | *SPAST* | 63 | 12 | c.1378C>T/- | Missense | Pathogenic^5^ | 45 | 14 | 6 | 1 |
| 5 | HSP12 | *SPAST* | 58 | 7 | c.1741C>T/- | Nonsense | Pathogenic^5^ | 14 | 16 | 8 | 3 |
| 5 | HSP13 | *SPAST* | 61 | 7 | c.1741C>T/- | Nonsense | Pathogenic^5^ | 54 | 2 | 7 | 2 |
| 6 | HSP14 | *SPAST* | 56 | 5 | **c.1145G>T/-** | **Missense** | **Likely pathogenic**  **(PM1,PM2,PP1,PP3)** | 32 | 24 | 29 | 4 |
| 6 | HSP15 | *SPAST* | 58 | 2 | **c.1145G>T/-** | **Missense** | **Likely pathogenic**  **(PM1,PM2,PP1,PP3)** | 42 | 17 | 23 | 4 |
| 7 | HSP16 | *SPAST* | 34 | 9 | c.1267G>T/- | Missense | Pathogenic^5^ | 23 | 11 | 22 | 3 |
| 7 | HSP17 | *SPAST* | 36 | 8 | c.1267G>T/- | Missense | Pathogenic^5^ | 29 | 7 | 8 | 2 |
| 8 | HSP18 | *SPAST* | 61 | 4 | **c.1360_1361insGGG/-** | **In-frame insertion** | **Likely pathogenic**  **(PM1,PM2,PM4,PP1,PP3)** | 55 | 6 | 23 | 4 |
| 8 | HSP19 | *SPAST* | 52 | 4 | **c.1360_1361insGGG/-** | **In-frame insertion** | **Likely pathogenic**  **(PM1,PM2,PM4,PP1,PP3)** | 40 | 12 | 15 | 3 |
| 8 | HSP20 | *SPAST* | 42 | 11 | **c.1360_1361insGGG/-** | **In-frame insertion** | **Likely pathogenic**  **(PM1,PM2,PM4,PP1,PP3)** | 10 | 32 | 16 | 3 |
| 8 | HSP21 | *SPAST* | 44 | 5 | **c.1360_1361insGGG/-** | **In-frame insertion** | **Likely pathogenic**  **(PM1,PM2,PM4,PP1,PP3)** | 25 | 19 | 18 | 3 |
| 9 | HSP22 | *SPAST* | 38 | 8 | c.1651G>C/- | Missense | Pathogenic^27^ | 30 | 8 | 16 | 2 |
| 10 | HSP23 | *SPAST* | 54 | 7 | c.1667_1668delCA/- | Frameshift | Pathogenic^27^ | 21 | 33 | 42 | 5 |
| 10 | HSP24 | *SPAST* | 61 | 4 | c.1667_1668delCA/- | Frameshift | Pathogenic^27^ | 30 | 31 | 19 | 4 |
| 11 | HSP25 | *SPAST* | 58 | 3 | c.1255G>T/- | Nonsense | Pathogenic^27^ | 37 | 21 | 26 | 4 |
| 12 | HSP26 | *SPAST* | 35 | 11 | c.1651G>C/- | Missense | Pathogenic^27^ | 24 | 11 | 18 | 3 |
| 12 | HSP27 | *SPAST* | 65 | 4 | c.1651G>C/- | Missense | Pathogenic^27^ | 37 | 28 | 17 | 3 |
| 13 | HSP28 | *SPAST* | 34 | 15 | c.1841C>T/- | Missense | Pathogenic^28^ | 14 | 20 | 12 | 3 |
| 14 | HSP29 | *SPAST* | 13 | 9 | c.1495C>T/- | Missense | Pathogenic^27^ | 2 | 11 | 8 | - |
| 14 | HSP30 | *SPAST* | 54 | 1 | c.1495C>T/- | Missense | Pathogenic^27^ | 0 | 54 | 8 | 2 |
| 15 | HSP31 | *SPAST* | 60 | 11 | **c.1531G>T/-** | **Nonsense** | **Likely pathogenic**  **(PVS1,PM2)** | 43 | 17 | 32 | 4 |
| 16 | HSP32 | *SPAST* | 72 | 8 | c.1651G>C/- | Missense | Pathogenic^27^ | 57 | 15 | 23 | 4 |
| 17 | HSP33 | *SPAST* | 43 | 11 | c.1291C>T/- | Missense | Pathogenic^29^ | 36 | 7 | 21 | 4 |
| 17 | HSP34 | *SPAST* | 64 | 4 | c.1291C>T/- | Missense | Pathogenic^29^ | 43 | 21 | 20 | 4 |
| 18 | HSP35 | *SPAST* | 50 | 12 | c.1493G>C/- | Missense | Pathogenic^30^ | 45 | 5 | 43 | 5 |
| 18 | HSP36 | *SPAST* | 43 | 11 | c.1493G>C/- | Missense | Pathogenic^30^ | 32 | 11 | 23 | 4 |
| 19 | HSP37 | *SPG11* | 35 | 12 | c.2444+1G>C/c.2444+1G>C | Splicing | Pathogenic^5^ | 15 | 21 | 44 | 5 |
| 20 | HSP38 | *SPG11* | 32 | 11 | c.1621C>T/c.7000G>C | Nonsense/Missense | Pathogenic^5^ | 20 | 14 | 41 | 5 |
| 21 | HSP39 | *SPG11* | 31 | 13 | c.733_734delAT/c.733_734delAT | Frameshift | Pathogenic^5^ | 14 | 18 | 35 | 5 |
| 22 | HSP40 | *SPG11* | 39 | 7 | c.433dupC/c.433dupC | Frameshift | Pathogenic^5^ | 18 | 22 | 28 | 5 |
| 22 | HSP41 | *SPG11* | 45 | 7 | c.433dupC/c.433dupC | Frameshift | Pathogenic^5^ | 20 | 27 | 38 | 5 |
| 23 | HSP42 | *CYP7B1* | 56 | 4 | c.889A>G/ c.961G>A | Missense/ Missense | Pathogenic^5^/ Pathogenic^5^ | 30 | 28 | 36 | 4 |
| 23 | HSP43 | *CYP7B1* | 47 | 6 | c.889A>G/ c.961G>A | Missense/ Missense | Pathogenic^5^/ Pathogenic^5^ | 30 | 18 | 18 | 3 |
| 23 | HSP44 | *CYP7B1* | 51 | 4 | c.889A>G/ c.961G>A | Missense/ Missense | Pathogenic^5^/ Pathogenic^5^ | 36 | 16 | 34 | 4 |
| 23 | HSP45 | *CYP7B1* | 62 | 4 | c.889A>G/ c.961G>A | Missense/ Missense | Pathogenic^5^/ Pathogenic^5^ | 40 | 23 | 45 | 4 |
| 24 | HSP46 | *SPG7* | 65 | 16 | c.1450-1_1457delGGAGAGGCG/ c.2014G>A | Frameshift/Missense | Pathogenic^5^/ Pathogenic^5^ | 34 | 32 | 21 | 4 |
| 25 | HSP47 | *SPG7* | 71 | 15 | c.1450-1_1457delGGAGAGGCG/ c.1715C>T | Frameshift/Missense | Pathogenic^5^/ Pathogenic^5^ | 22 | 50 | 34 | 4 |
| 26 | HSP48 | *SPG7* | 24 | 18 | **c.1997G>T/ c.1997G>T** | **Missense** | **Variant of unknown significance**  **PM2,PM5,PP3^c^** | 9 | 15 | 13 | 3 |
| 27 | HSP49 | *ATL1* | 26 | 12 | c.757G>A/- | Missense | Likely pathogenic^31^ | 12 | 14 | 6 | 1 |
| 27 | HSP50 | *ATL1* | 51 | 6 | c.757G>A/- | Missense | Likely pathogenic^31^ | 11 | 40 | 11 | 3 |
| 28 | HSP51 | *CYP27A1* | 49 | 5 | c.1421G>A/ c.1421G>A | Missense | Pathogenic^5^ | 20 | 10 | 30 | 5 |
| 29 | HSP52 | *CYP27A1* | 36 | 13 | c.1435C>G/c.1435C>T | Missense | Pathogenic^32^ | 30 | 6 | 9 | 2 |
| 30 | HSP53 | *CYP27A1* | 44 | 11 | NA^b^ | NA | NA | 1 | 43 | 40 | 3 |
| 31 | HSP54 | *CYP27A1* | 61 | 3 | NA^b^ | NA | NA | 50 | 11 | 4 | 0 |

**e-Table 1: Detailed individual data of HSP subjects**

Fam, family; NA, not available; SPRS, Spastic Paraplegia Rating Scale; ^a^ variant classification according to the 2015 American College of Medical Genetics and Genomics (ACMG) criteria, numbers after the variant classification indicates the reference that previously described this variant. Bold indicate novel variants. Each criteria of ACMG 2015 utilized to classify novel variant is given bellow the classification; ^b^ diagnosis of cerebrotendinous xanthomatosis was performed based on suggestive clinical findings and by marked elevation of plasma cholestanol levels; ^c^ this patient was included because his phenotype of spastic paraparesia, ataxia, and ophthalmoplegia was suggestive of SPG7 (although it was not possible to fulfill ACMG criteria PP4), because the variant c.1997G>T in *SPG7* was not found on gnomAD and 1000 genomes, the missense change occurred at an amino acid residue where a different pathogenic missense change has been seen before, and because it was predictive to be damaging by different in silico tools (M-CAP, CADD, Mutation taster, SIFT, PolyPhen-2). Variants were described with reference to the following transcripts: *ATL1* (NM_015915.4), CYP7B1 (NM_004820.3), CYP27A1 (NM_000784.3), SPAST (NM_014946.3), SPG7 (NM_003119.3), SPG11 (NM_025137.3) and reads were mapped to hg19.
