# Supplementary material for: Are Cognitive Changes in Hereditary Spastic Paraplegias Restricted to Complicated Forms?
Source: Front Neurol. 2019 May 24;10:508. doi: 10.3389/fneur.2019.00508 (PMC6558376; doi:10.3389/fneur.2019.00508)
Supplement: Supplementary file 2 [file Table_2.DOCX]

|  | **Truncating variants in *SPAST*** | **Non-truncating variants in *SPAST*** | **Statistics** |
| --- | --- | --- | --- |
| **Age (y)** | 55.45 (6.75) | 48.4 (12.61) | t(29)=1.716, P=0.097 |
| **Educational level (y)** | 6.36 (3.32) | 8.45 (3.95) | t(29)=1.483, P=0.149 |
| **Age at Onset (y)** | 40.27 (11.03) | 31.65 (18.82) | t(29)=1.387, P=0.176 |
| **Disease Duration (y)** | 15.45 (9.27) | 17 (12.54) | t(29)=0.357, P=0.724 |
| **SPRS** | 23.91 (9.8) | 18.3 (8.8) | t(29)=1.621, P=0.116 |
| **Cross-sectional Disease Progression** | 2.35 (1.86) | 1.80 (1.84) | t(29)=0.791, P=0.435 |
| **MMSE** | 26 (5) | 26 (5) | U=97, P=0.611 |
| **MOCA** | 19.7 (4.72) | 20.45 (5.18) | t(29)=0.411, P=0.684 |
| **FAS** | 19.15 (9.46) | 17.82 (9.89) | t(29)=0.369, P=0.715 |
| **FAS-cat** | 11.9 (5.33) | 11.55 (3.56) | t(29)=0.197, P=0.845 |
| **RAVLT** | 23.4 (8.85) | 25.73 (7.98) | t(29)=0.724, P=0.475 |
| **A6** | 4 (2.24) | 3.82 (2.4) | t(29)=0.210, P=0.835 |
| **A7** | 2 (3) | 3.5 (4) | U=87.5, P=0.359 |

**e-Table 2 – Genotype-phenotype correlation in SPG4**

Data are shown as means (standard deviation), except for MMSE and A7 that are shown as median (interquartile range). FAS, verbal fluency with phonological restriction; FAS-cat, verbal categorical fluency (animals); MMSE, Mini Mental State Examination; MOCA, Montreal Cognitive Assessment; RVALT, A6 and A7, Rey's Verbal Auditory Learning Test; SPRS, Spastic Paraplegia Rating Scale; y, years. Only adult patients with SPG4 were considered for this analysis.
